# New Challenges for Detection and Control of Foodborne Pathogens: From Tools to People

**DOI:** 10.3390/foods11121788

**Published:** 2022-06-17

**Authors:** Pilar Truchado, Walter Randazzo

**Affiliations:** 1 Research Group on Microbiology and Quality of Fruit and Vegetables, CEBAS-CSIC, 30100 Murcia, Spain; 2 VISAFE Lab, Department of Preservation and Food Safety Technologies, Institute of Agrochemistry and Food Technology, IATA-CSIC, 46980 Valencia, Spain

Contamination of foods by human pathogenic microorganisms is a major concern to both food safety and public health. The changes in consumers’ demand, the globalization of the food trade, and the progress on food production practices and processing technologies all pose new challenges for food industries and regulatory agencies to ensure the safety in food products.

With regard to microbiological safety, bacteria and viruses are the most common foodborne pathogens associated with both sporadic cases and outbreaks.

However, bacterial and viral microorganisms differ in terms of their behavior in food matrices, their stability in food-related environments (e.g., food-contact surfaces, irrigating and processing waters), and their response to food processing technologies and controlling measures. Current methods do not meet all relevant criteria for effective monitoring plans, the main limitations being their sensitivity, the high workload and time requirement, and the inability to differentiate between viable and non-viable microorganisms. Thus, specific and sensitive methods need to be developed for their detection and quantification in complex matrices, such as food, for tracking their occurrence along the food chain to determine the sources of contamination, and for ultimately estimating the risk for consumers.

To fill these gaps, this Special Issue comprises four original research articles and a review paper focusing on the implementation of novel analytical techniques and approaches to foodborne pathogens along the food chain.

Zand and colleagues [1] reviewed the most recent advances of the application of flow cytometry (FCM) and fluorescence in situ hybridization (FISH) for the rapid detection and characterization of microbial contamination. FCM allows for a culture-independent quantification of microbial cells, also providing information on their physiological and structural characteristics which are relevant to assess their viability status. FISH is a nucleic acid-based method mainly applied in the medical and diagnostic fields. While FCM has been successfully used to detect and monitor microorganisms in water, state-of-the-art FCM and FISH protocols for food matrices still show significant limitations. The main pitfalls include complex sample preparation steps; the use of toxic substances; their limits of detection, especially for FISH assays; and the equipment price. Because of all these aspects, FCM and FISH have not yet gained considerable interest in food safety area for the detection of microbial pathogens. Future studies should focus on potential optimization strategies for FCM and FISH protocols in food samples and their validation, as well as on the development of automated lab-on-chip solutions.

Moving to explore next-generation sequencing (NGS) applications in the produce industry, Truchado et al. [2] contributed to identify potential contamination niches of *Listeria monocytogenes* in a frozen vegetable processing plant. NGS is a sequencing technology that offers ultra-high throughput, a scalable and fast technique that allows the authors to characterize the isolates by a whole-genome sequencing (WGS) approach based on multi locus sequence typing (MLST). The WGS analysis revealed the presence of four different sequence types (ST) contaminating 18% of the samples, including food contact surfaces (FCS), non-food contact surfaces (n-FCS), and final product. These ST were further classified into four different virulence types (VT) according to multi-virulence locus sequence typing (MVLST). Interestingly, an isolate detected in non-food-contact surfaces (n-FCS) also contaminated the final product, highlighting the relevant role of n-FCS as reservoir of *L. monocytogenes* that reached the final product.

*Staphylococcus aureus* is a foodborne pathogen considered to be one of the etiological agents of food-related disease outbreaks. Leng et al. [3] supported this Special Issue with a study on its control using the skin mucus extract of Channa argus as a source of antimicrobial compounds. Of interest, untargeted metabolomics were applied to decipher its antibacterial mechanism against *S. aureus*. Results indicated that the extract had great inhibitory action on its growth by inducing the tricarboxylic acid cycle and amino acid biosynthesis, which are the primary metabolic pathways that affect the normal physiological functions of biofilms.

The present collection includes a second contribution on the control of *S. aureus* authored by Kim and colleagues [4] who developed a real-time PCR method (qPCR) for the rapid detection and quantification of pathogenic *Staphylococcus* species.

Four specific molecular targets were identified based on pan-genome analysis, and results showed 100% specificity for 100 non-target reference strains with a detection limit as low as 10^2^ CFU/mL. Thus, the proposed method allows an accurate and rapid monitoring of *Staphylococcus* species and may help control staphylococcal contamination of food.

Moving to human viral pathogens, Macaluso et al. [5] reported the results of an investigation aimed to characterize the occurrence of human enteric viruses in shellfish, a food item with relevant risk for consumers. The study included data collected over two years on the prevalence of enteric virus contamination along the shellfish production and distribution chain in Sicily, Italy. The findings based on quantitative reverse transcription polymerase chain reactions (RT-qPCRs), as gold-standard molecular technique, showed that almost 6% of samples were contaminated with at least one enteric virus such as norovirus, hepatitis A virus, and/or hepatitis E virus. The origin of contaminated shellfish was traced back to Spain and several municipalities in Italy. Such contribution highlights the relevance of routine monitoring programs to prevent foodborne transmission of enteric viruses and preserve the health of consumers.

In summary, this Special Issue compile several contributions focused on novel technologies, approaches, and strategies demonstrated to be effective in controlling microbial contamination in food. All the articles provide valuable information to monitor and/or reduce contamination in food, food industry settings, and along the food chain.

On a final note, the collection emphasizes the relevance of ensuring food safety and limiting the risk of microbiological contamination along the food chain to protect consumers.

## Data Availability

Not applicable.

## References

[1] Zand E., Froehling A., Schoenher C., Zunabovic-Pichler M., Schlueter O., Jaeger H. (2021). Potential of Flow Cytometric Approaches for Rapid Microbial Detection and Characterization in the Food Industry—A Review. Foods.

[2] Truchado P., Gil M.I., Querido-Ferreira A.P., Capón C.L., Álvarez-Ordoñez A., Allende A. (2022). Frozen Vegetable Processing Plants Can Harbour Diverse *Listeria monocytogenes* Populations: Identification of Critical Operations by WGS. Foods.

[3] Leng W., Wu X., Shi T., Xiong Z., Yuan L., Jin W., Gao R. (2021). Untargeted Metabolomics on Skin Mucus Extract of *Channa argus* against *Staphylococcus aureus*: Antimicrobial Activity and Mechanism. Foods.

[4] Kim E., Yang S.-M., Won J.-E., Kim D.-Y., Kim D.-S., Kim H.-Y. (2021). Real-Time PCR Method for the Rapid Detection and Quantification of Pathogenic *Staphylococcus* Species Based on Novel Molecular Target Genes. Foods.

[5] Macaluso G., Guercio A., Gucciardi F., Di Bella S., La Rosa G., Suffredini E., Randazzo W., Purpari G. (2021). Occurrence of Human Enteric Viruses in Shellfish along the Production and Distribution Chain in Sicily, Italy. Foods.

